# Formulary Management Activities and Practice Implications Among Public Sector Hospital Pharmaceutical and Therapeutics Committees in a South African Province

**DOI:** 10.3389/fphar.2020.01267

**Published:** 2020-08-18

**Authors:** Moliehi Matlala, Andries G. S. Gous, Johanna C. Meyer, Brian Godman

**Affiliations:** ^1^ School of Pharmacy, Sefako Makgatho Health Sciences University, Ga-rankuwa, South Africa; ^2^ Strathclyde Institute of Pharmacy and Biomedical Sciences, University of Strathclyde, Glasgow, United Kingdom; ^3^ Department of Laboratory Medicine, Division of Clinical Pharmacology, Karolinska Institutet, Karolinska University Hospital Huddinge, Stockholm, Sweden

**Keywords:** formulary, management, essential medicines, rational medicine use, list (data structure), pharmaceutical and therapeutics committees, South Africa

## Abstract

**Introduction:**

The World Health Organization identified Pharmaceutical and Therapeutics Committees (PTCs) at district and hospital levels as one of the pivotal models to promote rational use of medicines (RUM). This is endorsed by the Government in South Africa. Formulary development and management is one of the main functions of PTCs. This study aimed to describe the formulary management activities among PTCs in public hospitals in Gauteng Province, South Africa, following initiatives to promote RUM in South Africa.

**Methods:**

Qualitative, nonparticipatory, observational study, observing 26 PTC meetings. Data were coded and categorized using NVivo9^®^ qualitative data analysis software. Themes and sub-themes were developed. The themes and sub-themes on formulary management are the principal focus of this paper.

**Results:**

More than half of the observed PTCs reviewed their formulary lists. There was variation in the review process among institutions providing different levels of care. Various aspects were considered for formulary management especially requests for medicines to be added. These included cost considerations (mainly focusing on acquisition costs), evidence-based evaluation of clinical trials, patient safety, clinical experience and changes in the National Essential Medicines List (NEML). The tertiary PTCs mostly dealt with applications for new non-EML medicines, while PTCs in the other hospitals mainly requested removal or addition of EML medicines to the list.

**Conclusion:**

This is the first study from Gauteng Province, South Africa, reporting on how decisions are actually taken to include or exclude medicines onto formularies within public sector hospitals providing different levels of care. Various approaches are adopted at different levels of care when adding to- or removing medicines from the formulary lists. Future programs should strengthen PTCs in specialized aspects of formulary management. A more structured approach to formulary review at the local PTC level should be encouraged in line with the national approach when reviewing possible additions to the NEML.

## Background 

Access to and affordability of medicines is a challenge especially in low- and middle-income countries (LIMCs) including South Africa (Cameron et al., 2009; Leisinger et al., 2012; Attaei et al., 2017; Wirtz and Moucheraud, 2017; Chigome et al., 2019). One way to address this is to improve the appropriate use of medicines, given that more than half of all medicines are prescribed or dispensed inappropriately with approximately half of all patients failing to take their medicines correctly (World Health Organization, 2012; Ofori-Asenso and Agyeman, 2016). Appropriate use of medicines also reduces the extent of adverse drug reactions with their impact on morbidity, mortality and costs (Davies et al., 2009; Chiatti et al., 2012; Gyllensten et al., 2013; Formica et al., 2018). One recognized way to improve medicine use across LMICs is to ensure access to, and availability of, a core list of well proven and cost-effective medicines alongside measures to enhance universal healthcare, which was the concept behind the WHO’s Essential Medicines List (EML) (WHO Policy Perspectives on Medicines, 2002; World Health Organisation, 2015; Wirtz et al., 2017; Holloway et al., 2020).

The WHO’s recommendations for essential medicines programs encourage the use of formularies, as essential tools for the appropriate, safe and cost-effective use of medicines in order to promote the rational use of medicines (RUM) (Gustafsson et al., 2011; Schiff et al., 2012; Quick et al., September 2002). Formularies are seen as important for delineating and directing appropriate prescribing enhanced by trust in their development (Rucker and Schiff, 1990; Pillans et al., 1992; Eriksen et al., 2018). This is especially important when the principal means of physician education is *via* pharmaceutical companies (Spurling et al., 2010; Civaner, 2012; Jacob, 2018; Fadare et al., 2018a; Fadlallah et al., 2018).

The WHO identified Pharmaceutical and Therapeutics Committees (PTCs), also referred to as Drugs and Therapeutics Committees (DTCs) in a number of countries (Bjorkhem-Bergman et al., 2013; Hoffmann, 2013), at district and hospital levels as one of the pivotal models to promote RUM (Holloway and Green, 2003; Office of Director of Pharmaceutical Services (ODPS) Ministry of Health Ghana, 2015). Furthermore, a limited list of medicines for procurement, based on an EML or formulary, helps define which medicines should be regularly purchased and prescribed. Consequently, PTCs are seen as one of the most effective ways to control medicine usage and expenditure (Norman et al., 2009; Bjorkhem-Bergman et al., 2013; Tseng et al., 2016; Shirneshan et al., 2016; Meyer et al., 2017; Leporowski et al., 2018).

Formulary development and management is one of the key functions of PTCs (Holloway and Green, 2003; Hoffmann, 2013; Plet et al., 2013; Lima-Dellamora Eda et al., 2014; Matlala et al., 2017). A PTC can also provide the leadership and structure needed to select appropriate medicines for inclusion in the formulary, promote RUM, educate physicians on evidence-based medicine (EBM) and reduce waste, thereby optimizing medicine expenditure and improving patient outcomes within available resources (Wu and Miao, 2008; Bjorkhem-Bergman et al., 2013; Hoffmann, 2013; Lima-Dellamora Eda et al., 2014). Decisions for the listing of medicines on formularies, including new medicines, should be based on clinical, ethical, legal, social, philosophical, quality-of-life, safety and economic factors (Holloway and Green, 2003; Jenkings and Barber, 2004; Tyler et al., 2008; Puigventos et al., 2010; Gustafsson et al., 2011; Hoffmann, 2013; Plet et al., 2013). Decisions should also take into account issues such as access and implementation as well as follow-up and procurement (Pharasi and Miot, 2013; Lima-Dellamora Eda et al., 2014; Meyer et al., 2017; Matlala et al., 2017). In addition, local knowledge should be taken into consideration, although decisions should be based on scientific rationality (Jenkings and Barber, 2004; Bjorkhem-Bergman et al., 2013).

There has been considerable development toward universal healthcare in South Africa in recent years in preparation for the implementation of National Health Insurance (NHI) to address previous inequalities for the majority of patients in South Africa (Meyer et al., 2017). Within this system, public hospitals are funded on an activity basis which includes the costs of medicines. Consequently, recommendations for prescribed medicines need to follow a rigorous and encompassing process to optimize the use of available resources (Meyer et al., 2017). The use of EBM as a key criteria for the selection of medicines for their inclusion into the Standard Treatment Guidelines and Essential Medicines List (STGs/EML) in South Africa has become robust and stringent with time (Republic of South Africa, 2018; National Department of Health, 2019). The basic criteria for medicines selection in South Africa is stipulated in the National Drug Policy, with the EML as the foundation for the development of STGs aimed at improving patient care (South African National Department of Health, 1996). The STGs/EML are regularly updated and available electronically to enhance their use (Meyer et al., 2017).

**Figure 1:**
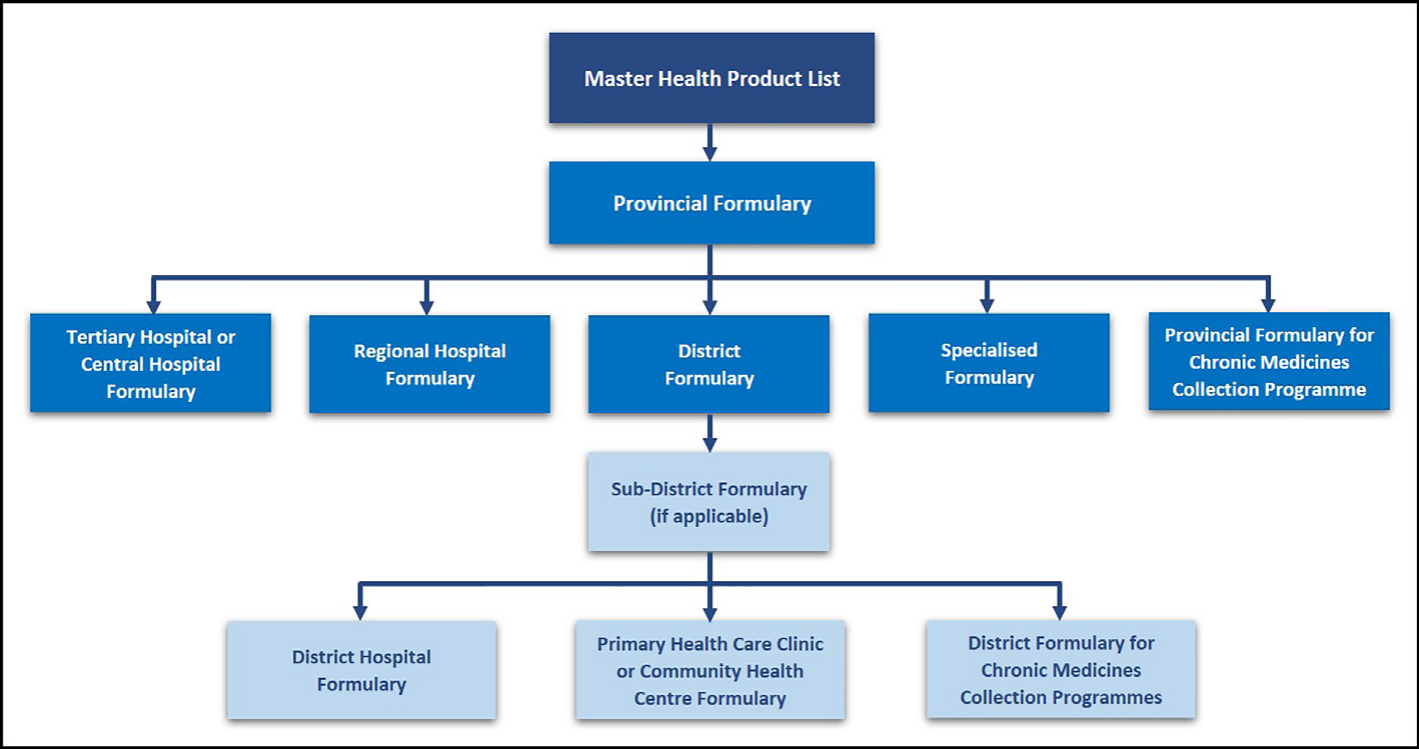
Hierarchy of Formulary Development and Management in the Public Sector – adapted from (South African National Department of Health, 2019).

The selection of medicines that are available in the public healthcare sector currently takes place through the National Essential Medicines List Committee (NEMLC) as well as provincial and facility based PTCs (Meyer et al., 2017; Republic of South Africa, 2018). Two essential tasks of PTCs within the public healthcare system are to develop and revise institutional STGs (usually adapted from national guidelines) and to maintain an institutional formulary, based on the national EML (Laing et al., 2001; Holloway and Green, 2003; Fadare et al., 2018b). The formulary development process follows a hierarchal approach according to the level of care, as outlined in the National PTC guidelines (South African National Department of Health, 2019). The Master Health Product List, which contains both EML and non-EML medicines, is the main document used to derive the respective institutional or level of care formulary lists as demonstrated in **Figure 1**. Tertiary level PTCs can evaluate potential non-EML medicines for inclusion in the hospital formulary; with secondary care and other hospitals confined to the EML in their deliberations.

In addition, PTCs monitor drug utilization against agreed guidance (Bjorkhem-Bergman et al., 2013; Eriksen et al., 2017; Leporowski et al., 2018). The PTCs within hospitals or from primary healthcare (PHC) facilities through their district PTCs, which are considered as Expert Review Committees, subsequently make recommendations for new medicines to be included in the STGs/EML (Matlala et al., 2017; Meyer et al., 2017; Mashaba et al., 2019).

The National Policy for the Establishment and Functioning of PTCs in South Africa outlined the standards for PTCs at all levels in South in 2015 (NDOH, 2015). The Policy also stipulates that PTCs should exist at provincial, district, sub-district (where applicable) and health establishment (Regional, Tertiary, Central and District Hospital) with, as mentioned, different degrees of freedom depending on their level of specialization. Consequently, the number of PTCs in each Province depends on the number of hospitals and districts. The composition and functions of PTCs in public sector hospitals within the Gauteng Province have been discussed in previously published studies (Matlala et al., 2017; Mashaba et al., 2019), including the number of meetings per annum, the activities carried out by these PTCs, the types of subcommittees within the PTC and their degree of freedom (NDOH, 2015; Matlala et al., 2017). The National and the Gauteng Provincial guidelines for PTCs in South Africa stipulate that PTCs should develop a formulary aligned to agreed treatment guidelines and protocols, and subjected to robust evidence-based interrogation (Gauteng Province Department of Health, 2013; NDOH, 2015). This process of evaluation must be conducted using explicit documented criteria, allowing for transparent evaluation of the decision and facilitating any reviews. This includes information on the pharmacological action of the medicine as well as its health gain versus current medicines on the formulary and the strength of the evidence (Gauteng Province Department of Health, 2013), with applications made on official forms.

Even though PTCs have been active in South Africa since the mid 1990s, little is known about the actual formulary management within South African public sector hospitals (Matlala et al., 2017; Perumal-Pillay and Suleman, 2017). This study aims to describe formulary management practices in public sector hospitals in the Gauteng Province of South Africa, and to recommend strategies to improve formulary management by PTCs in this province and wider, should this be necessary. This builds on our recent publications describing ongoing processes in the public health system in South Africa to improve the quality and efficiency of medicine use (Meyer et al., 2017), as well as the structure and functioning of PTCs among public sector hospitals (Matlala et al., 2017; Mashaba et al., 2019).

## Methods

A qualitative, nonparticipatory, observational method was employed for this study. Data collection entailed the observation of a total number of 26 PTC meetings (amounting to at least two meetings per PTC), conducted by 13 PTCs in the Gauteng Province, over a period of 12 months from February 2013 to February 2014. Requests to attend each of the scheduled PTC meetings were sent to all the responsible pharmacists at the hospital level, the district pharmacist and the chairperson of the provincial PTC *via* electronic mail. A letter detailing the objectives of the study, proof of ethical clearance and evidence of provincial approval were included in the communication.

In the instances where the chairperson of the PTC did not allow recording of the proceedings, notes of the discussions were taken and subsequently verified against the minutes of the meeting from the secretariat of the particular PTC. However, this was the case for only the Provincial PTC meetings. Observations were undertaken as discreetly as possible so as to not influence the proceedings of the PTC meetings. The observations were conducted until data saturation was reached.

The recorded data were transferred from the digital voice recorder to a computer and stored as Windows Media Audio files. The recording of each observed PTC meeting was transcribed verbatim, typed in Microsoft Word® and stored as a separate document. The transcribed observations were then verified against the audio recordings for accuracy, after which they were imported into NVivo® (Version 9) a software program for qualitative data analysis. Typed-up observational notes of PTC meetings which were not audio recorded, were also imported into NVivo®.

Two of the authors (MM and JCM) performed a thematic analysis of the data using open coding (**Table 1**) (Braun and Clarke, 2006). The transcripts and observational notes were first read and re-read to explore and obtain an understanding of the data. A list of possible codes was compiled from the initial reading of the data, which were used as the starting point for coding with the software. Through consensus discussions between the two coders, code-recoding procedures were employed to increase the consistency (dependability) of the findings. Connections between codes were identified and categories created. Additional codes and categories were created as new topics emerged. The categories were reviewed and discussed with the other co-authors for consensus of coding and emergent themes and subthemes. The themes and subthemes specifically relating to formulary management were identified as the focus point for this paper.

**Table 1:** Data analysis process.

Steps of analysis	Tasks completed
**Step 1:** Data familiarization	Transcription, verification of transcripts against audio recordings and field notes, as well as re-reading of transcripts
**Step 2:** Initial codes development	Initial open coding of the entire data set
**Step 3:** Identification of theme	Categorization of codes into potential themes
**Step 4:** Review of themes	Confirming themes - ensuring the internal homogeneity and external heterogeneity of themes
**Step 5:** Defining and naming of themes	Further refinement of themes, specifically identifying those that relate to formulary management
**Step 6:** Report finalization	Production of the manuscript, selection of illustrative quotes

The results are presented according to the themes and sub-themes that emerged from the data with verbatim quotations of discussions from PTC members inserted to support the findings. A coding system was used to protect the identity of the participating PTCs and its members. Quotations were credited to specific PTCs and participants by using a dedicated reference code for the observation of a particular PTC according to the level of care and the meeting observed. Quotations are presented in italics and enclosed in inverted commas. Identifiers of the participants are provided in square brackets after each quotation to enhance understanding. Explanations included in the quotations are presented as normal text and enclosed in round brackets. **Table 2** provides details of the codes assigned to PTC observations.

**Table 2:** Reference codes assigned to Pharmaceutical and Therapeutics Committee (PTC) meeting observations.

		Reference code
**Data collection method**	PTC Observation	Ob
**Level of PTC**	Provincial	P
	District	D
	Tertiary Hospital	Th
	Regional Hospital	Rh
	District Hospital	Dh
**Example** **‘Quotation’ **	Notes taken from observation of PTC meeting at a tertiary Hospital	[Ob Th PTC]

### Ethics Statement

The Medunsa Research Ethics Committee of the University of Limpopo (Medunsa Campus), now Sefako Makgatho Health Sciences University, granted ethical clearance for the study (MREC/H/170/2010:PG). Approval from the Gauteng Provincial Department of Health Research Committee was obtained prior to the study implementation. Chairpersons of the respective PTCs provided permission to record or take notes of proceedings.

## Results

### Thematic Analysis of Content

The thematic analysis of the content of the observational transcripts resulted in seven major themes and subthemes, which are summarized in **Table 3**.

**Table 3:** Summary of themes and subthemes relating to formulary management.

Category	Themes and subthemes
**Inputs**	Application of evidence-based medicine principles
	Patient benefit Patient safety
	Cost considerations
	Cost minimization analysis Estimated annual expenditure
**Resources**	Expertise and experience
	Pharmacoeconomic Expertise Clinical Experience
**Controls**	Internal and external controls
	EML/STG reviews Provincial guidelines
**Outputs**	Acceptance of application
	Cost savings Restricted patient use Conditional up-referral of decision to management Development of clinical guidelines for accepted medicine
	Decline of application
	Requested medicine not registered for indication Insufficient evidence from data presented
**Updates**	Removal of medicines from formulary list
	Decreased prescribing EML changes Medicine shortages Level of care Cost of medicines

#### Theme 1: Application of EBM Principles

When an application for new medicines outside of the current hospital formulary list, and in some cases outside the EML, were presented at the PTC, either the chairperson or the secretariat first introduced the request. The clinician who submitted the request, if present at the meeting, was subsequently asked to present the rationale behind the request. In the presentations at tertiary and regional PTCs, the clinicians typically provided a summary of a clinical trial that demonstrated the benefits of the medicine being requested for inclusion. This was typically not the case in secondary and primary care hospitals.

##### Subtheme 1a: Patient Benefit

The common features of the evidence presented were the comparison of a new medicine to an existing medicine in a Cochrane review (e.g. balanced salt solution). Survival rates were also referred to in some instances (e.g. for a proposed oncology medicine). In the case of a proposed oncology medicine, several clinical trials were presented to indicate an improvement in survival. In other instances, clinical trial information was used to demonstrate improved morbidity.

“*Diffuse large B cell lymphomas are potentially curable and pre-treatment variables do predict for prognosis and response to treatment. With an IPI score of zero there is about a 94% survival at 4 years, if the patients are treated with a standard chemotherapy including rituximab, and a lower IPI score then there is about 50% survival at five years”.* [Ob Th PTC]

It was evident that the principles of EBM were used in decision-making at tertiary hospital PTCs. The clinician also discussed the outcomes of treatment when an alternative management approach was compared to the use of the medicine being considered. This is illustrated by an example where the results of using radiation alone, instead of using the anticancer medicine, was highlighted.

*“Results in the treatment of diffuse large B cell lymphomas, if untreated survival can be measured in months. Radiation therapy has been looked at in diffuse large B cell lymphomas, but recurrence occurs at a rate of 70%, so radiation therapy is not first line treatment or the second line treatment and only in certain cases is it indicated as consolidation therapy”.* [Ob Th PTC]

The discussion was further informed by highlighting the overall survival rate when using the medicine being requested.

*“They were able to demonstrate that rituximab together with CHOP resulted in significant improvement in overall survival at 7 years and event free survival of about 32% and a 10 years overall survival of 44%”*. [Ob Th PTC]

##### Subtheme 1b: Patient Safety

The PTCs in secondary and other hospitals also considered EBM principles in submissions, though to a lesser extent. The main considerations for including new medicines into hospital formularies were relative risk reduction, clinical benefits and patient safety.

The relative risk or side effect profiles of the new medicine were mentioned with reference to international commentary, as illustrated below.

*“There was no difference between efficacy and side effects when used in children. Although in this European comment they say that zelondronic acid may result in some minor complications like the inflammatory type of reaction, unexplained tachycardia, acute phase response and decreased calcium levels, are more prominent with the use of zelondronic acid. The side effects are often relative”.* [Ob Th PTC]

In some instances, patient safety was also a motivation when medicines were removed from the formulary.

*“Phenytoin IV is not safe to use at the primary care level because of the required monitoring, other anticonvulsants can be used to control patients that present at the primary clinic with a fit and the patient can then be transferred to the hospital once controlled. Oral anticonvulsants can be given to patients that can swallow which are much safer than the IVI; the IVI can be given at the hospitals where monitoring takes place”.* [Ob D PTC]

In all cases, presentations were followed by questions from committee members and subsequent discussion, before a decision was taken to accept or reject the request.

#### Theme 2: Cost Considerations

Cost was one of the main considerations when new medicines were requested for addition to the formulary at all levels of care. The cost was typically referred to as the cost per dose, cost per patient, or an estimate of the total annual drug expenditure. At the time of this study, there was little consideration of formal health economic argumentation such as cost per quality adjusted life year.

##### Subtheme 2a: Cost-Minimization Analysis

There was a difference in the cost considerations with regard to the hospital level. Most new medicine considerations at tertiary level hospital PTCs were mainly for non-EML medicines. In such instances, the acquisition costs of the new medicines were compared to similar agents in the hospital. In some cases, additional costs, such as mode of administration and costs of other monitoring measures were also considered.

*“The cost of Actilyse® is about R3 000.00 or R4 000.00 per vial, which is marginally less expensive than the cost of Metalyse®, but Metalyse® is more effective as there are no infusion pumps and drips sets, so it ends up working out to be much cheaper”*. [Ob Th PTC]

##### Subtheme 2b: Estimated Annual Expenditure

Estimated annual expenditure was often referred to when the proposed new medicine was going to be used in only a few patients.

*“It is terribly expensive, and this is a lifelong treatment, this was discussed with the finance officer. Often acromegaly patients can be treated with up to 1mg per day; the LAR is 20mg monthly, which works out to R21 000.00 per month. The yearly cost is more than R210 000.00”.* [ObTh PTC]

Similarly, the cost per patient was often used in discussions, especially when the acquisition cost of the proposed medicine was high.

*“In severe over reactive bladder they need botox intravisicle once off and it is then repeated once a year. It is a very important problem. The cost will be R1 950.00 per patient that adds up to R4 000.00 per year for the two patients. It might be repeated after one year”.* [Ob Th PTC]

#### Theme 3: Expertise and Experience

##### Subtheme 3a: Pharmacoeconomic Expertise

There was a perceived lack of expertise in pharmacoeconomic evaluations among PTC members. In one of the observations, a member of the tertiary level PTC highlighted the fact that they lacked expertise in undertaking or fully assessing a formal economic evaluation when considering whether to list new medicines onto the formulary. This is an area to address in the future.

*“One of the things that are needed in the hospital is a health economist, because the committee is looking at just the drug costs as opposed to looking at all the other costs. If a cost-minimisation analysis was done, one might find that it is cheaper to give the medicine than treating the heart failure with all the other things the patients get”.* [Ob Th PTC]

##### Subtheme 3b: Clinical Experience

When a new medicine was being proposed especially for a non-EML medicine, the indication of the medicine was outlined and the clinician sometimes highlighted his/her clinical experience with the use of the particular medicine, e.g. a new treatment of type B cell lymphoma:

*“Diffuse large B cell lymphomas are potentially curable and pre-treatment variables do predict good prognosis and response to treatment. With an IPI score of zero there is about a 94% survival at 4 years, if the patients are treated with a standard chemotherapy, including rituximab and a lower IPI score then there is about 50% survival at five years”.* [Ob Th PTC]

In some instances, clinicians did not make specific reference to clinical trials. In these situations, they alluded to evidence that had demonstrated clinical benefit to the patient. The following observation illustrates how in this case the clinician used the experience from a tertiary hospital as a point of reference for the request:

*“This has become the procedural sedation of choice in high risk patient population groups, which is why in ICU use it gives a very different quality of sedation. Patients are clear, they are not as sedated as they are with benzodiazepines, where there is often difficulty with differentiating between delirium and nondelirium state. There is often synergism between benzodiazepines and other agents at the Gama Amino Butyric Acid receptor site that leads to marked haemodynamic instability intra-operatively. These medicines are now currently being used worldwide to perform these types of procedures specifically in high risk patient population groups”.* [Ob Rh PTC]

#### Theme 4: Internal and External Controls

##### Subtheme 4a: EML/STG Reviews

At district hospitals, discussions typically focused on formulary reviews. The pharmacists in the PTCs typically presented most of these discussions and submissions. The reviews were based on EML medicines, which needed to be included or removed from the formulary list. Some of the discussions were based on clinicians requesting medicines which were already on the EML but not available in their respective institutions. In these instances, the cost of the requested medicine was typically compared to a similar agent that was already being used in the hospital to justify inclusion, as illustrated by the following example:

*“The pharmacy received a request for captopril, as it is on the EML. However, the price of a packet of 56 captopril 25mg tablets is R6.79 and the price of 28 enalapril tablets is R2.60, and packet of 28 tablets perindopril costs R14.91”.* [Ob Dh PTC]

In some cases, the use of a different formulation was encouraged, also based on cost, as demonstrated with the use of risperidone tablets:

*“Risperdal® (Risperidone) 1mg tablets versus the syrup - the pharmacy requested that patients where possible be prescribed the tablets instead of the syrup, because the tablets’ tender price is R8.95 per packet whereas the syrup costs R181.00 per bottle”.* [Ob Dh PTC]

##### Subtheme 4b: Provincial Guidelines

The fact that formulary reviews at district hospitals was determined by the provincial formulary can be confirmed by one of the observations from the provincial PTC:

*“There are items that need to be removed from the local formulary in order to be in line with the provincial formulary”.* [Ob P PTC]

Provincial treatment guideline updates influenced decisions for removal of medicines in district/ secondary hospital PTCs, as illustrated below.

*“It was decided to remove the following medicines: griseofulvin tablets because it is not in the guidelines”.* [Ob D PTC]

Formulary decision-making for new proposed medicines as observed in this study were either accepted or declined. The decisions were made in consultation with the entire committee following rigorous discussions among the PTC members.

#### Theme 5: Acceptance of Application

##### Subtheme 5a: Cost Savings

Perceived cost savings that could be achieved was one of the motivating factors in PTCs accepting a request for a new medicine to be included in the hospital formulary. An observed example was for an ophthalmic preparation, where the proposed medicine was identified as cheaper than the alternative currently being used at the hospital, even though it was equally efficacious. The committee accepted the request based on lower acquisition costs.

*“The other motivation from ophthalmology is for Alphagan® (bromonidine) it is indicated for glaucoma, specifically for patients not responding to available treatment. The patients would be on one of the drugs available, if one wants to defer surgery an alpha-2 agonist can then be used. The cost for Betagan® (levobunolol) in this class is the lowest. The patients seen can be about 20 a year, but there is also the option of surgery. If the patient is on Trusopt® (dorzolamide) and Alphagan® is added on then it becomes expensive, but if the prescriber were to stop the Trusopt® and then add the Alphagan® it would not make a difference because they cost the same. It is approved for 20 patients per year; it will be useful to give feedback to the PTC regarding patient progress”.* [Ob Th PTC]

##### Subtheme 5b: Restricted Patient Use

Proposals were sometimes accepted for restricted patient use, but only where the patient population was clearly defined.

*“It would have to be stipulated that these agents will be reserved for high risk patient groups in theatre and for patients from cardiology coming for vascular procedures which are done noninvasively”.* [Ob Th PTC]

##### Subtheme 5c: Conditional Up-Referral of a Decision to Management

Some of the proposed new medicines were referred to the hospital administrators to decide whether to purchase or not, especially medicines which are perceived to be too expensive, following PTC support, based on clinical reasons. The PTC agreed that the medical indication exists, it was then up to the hospital’s management (referred to as the office of the Chief Executive Officer [CEO]) to decide if there is money for acquisition of the medicine.

*“It will have to go to the CEO’s office; the committee will write a letter that states there is clinical indication for the medicine”.* [Ob Th PTC]

*“It will have to go to the CEO’s office; the committee will write a letter stating the fact that there is a clinical indication for the medicine”. *[Ob Th PTC]

##### Subtheme 5d: Development of Clinical Guidelines for the Accepted Medicine

The observations of PTC meetings showed that some of the PTCs dealt with treatment guidelines in their meetings following a decision to accept the new medicine onto the hospital formulary. Clinicians were requested to develop guidelines for nonformulary medicines that could subsequently be approved by the committee, the aim being to control the prescribing of these medicines once on the formulary. In all instances where guideline development was required, the PTC did not outline the detail required in the guidelines. However, typically guidelines were needed before the introduction of a new medicine as illustrated by the discussion regarding the use of Precedex® (dexmedetomidine hydrochloride).

*“The committee will have to develop guidelines on how to control the use of Precedex®, and outline the patients who will be eligible for the agent”. [Ob Rh PTC]*

#### Theme 6: Decline of an Application

Various reasons were identified for PTCs declining requests.

##### Subtheme 6a: Requested Medicine Not Registered for the Indication

Some requests were declined because the medicines requested were not registered for the specific indication. This was despite the relative low cost compared to a medicine that was already being used for the particular indication. The following decision was taken by the tertiary hospital PTC, illustrating a case where the request was not accepted:

*“The challenge is when the motivation is for a medicine that is not registered for a disease when there is already a registered product. The concern is if the PTC approves the motivation and the medicine is used and something happens to the patient then, who takes responsibility for that. Clinical data should be provided along with the motivation”.* [Ob Th PTC]

##### Subtheme 6b: Insufficient Evidence From Data Presented

Some requests were rejected by a designated reviewer in the PTC based on a review of clinical trial data when it was felt that there was no demonstrated benefit for the indication/ patient population that the medicine was being considered for. The reviewer’s explanation during a tertiary level hospital PTC meeting illustrates the decision taken.

*“There are reasons why it is not used in these patients, because there are severe side effects which include 33% incidence of bone pain. There are quite a few papers on pneumonia, whatever type CAP, and multilobar. None of the papers unequivocally says that there is reduced mortality in these patients. In all these publications there is no conclusion to say that in severe pneumonia there will be reduced mortality”.* [Ob Th PTC]

#### Theme 7: Removal of Medicines From Formulary List

##### Subtheme 7a: Decreased Prescribing

The review of the list of medicines during the PTC meetings also involved removal of medicines, which in some instances was based on low usage. Such decisions were mostly undertaken at district hospital PTCs, with the presentations to PTCs mainly undertaken by the pharmacist. The following examples illustrate this approach:

*“Clomipramine tablets - the pharmacy suggested the decoding of clomipramine tablets due to the reduced usage*.” [Ob Dh PTC]

##### Subtheme 7b: EML Changes

Observations of provincial PTCs revealed that they are involved in an extensive review of all levels of the EML, which resulted in the removal of a number of medicines from the formulary provincial list.

*“In total there are now 366 items on the list. 71 items were removed, 46 items were added. Cimetidine was discussed an example of an item that had been removed because it is more costly than ranitidine and it has many interactions”.* [Ob P PTC]

In the case of glipizide, the primary care PTC recommended removal of glipizide from the Gauteng formulary:

*“The medicine (glipizide) is not included in the EML and usage in the province is minimal”.* [Ob P PTC]

##### Subtheme 7c: Medicine Shortages

The unavailability of medicines due to manufacturing challenges such as the unavailability of raw materials was also observed as one of the reasons for removal of medicines from the formulary, as illustrated below.

*“Nitrofurantoin capsules – ‘there has been a raw material problem for over 1 year, it should therefore be removed’.”* [Ob Dh PTC]

##### Subtheme 7d: Level of Care

Another factor that was considered when removing medicines from the formulary list was the level of care. As mentioned, different levels of PTCs serve healthcare facilities that provide different services. Consequently, the medicines that should be stocked at each level of care should be specific to the service provided, as outlined in the EML. The example below was at the district level of care, which is only allowed to keep medicines listed in the primary care EML.

*“Diclofenac injection was introduced as a result of a request by family physicians; however, the district was instructed to remove it from its formulary list as it is not on the primary EML”.* [Ob D PTC]

##### Subtheme 7e: Cost of Medicines

At provincial levels cost as consideration when reviewing the formulary list was as discussed

*“The review should be evidence based and cost needs to be brought into consideration through a cost effectiveness analysis.”* [Ob P PTC]

The cost of medicines was also considered when considering the removal of a medicine from the formulary list.

*“Hydrocortisone cream will be kept in place of the ointment due to price considerations; it was decided to remove the ointment”.* [Ob D PTC]

## Discussion

One of the main functions of the PTC is to ensure RUM through formulary management. In this study, we explored the process followed by PTCs in the Gauteng Province in performing this function. It was noteworthy and encouraging that the majority of PTCs in this study reviewed their formulary lists on an ongoing basis in line with American Society of Health-System Pharmacists (ASHP) guidelines (Tyler et al., 2008). This despite the fact that they were mainly limited to the EML and to an extent the tertiary medicines lists in the case of tertiary hospital level PTCs, as at the time the study was conducted the tertiary list had been newly introduced. While as expected among District Hospital and PHC level PTCs, there were no applications for non EML medicines, there was a level of interrogation of available medicines including adding new medicines from the EML list. The tertiary level PTCs were the ones who tried to introduce newer medicines as they had the advantage of access to tertiary and quaternary medicine lists, reserved for specialists at these hospitals. Even in such instances the high quality evidence of the medicine’s efficacy and effectiveness was required. Typically, clinicians who requested the new medicine onto the formulary were often present during the discussion of their application, which provided an opportunity to clarify any queries. This contrasts with Spain where applicants did not take part in the evaluation process for new medicines in 57% of cases (Duran-Garcia et al., 2011). We would certainly endorse clinician involvement more widely where possible, especially as this enhances formulary adherence as seen for the ‘Wise List’ in Sweden (Gustafsson et al., 2011; Bjorkhem-Bergman et al., 2013; Eriksen et al., 2017). In instances where high cost medicines were being requested principally at the tertiary level PTCs, more thorough evaluations were undertaken. These included analysis of the efficacy, effectiveness and cost considerations. This practice is similar to formulary management processes carried out in the private sector by medical aid schemes in South Africa (Pharasi and Miot, 2013). Among Danish hospitals, criteria used for inclusion of medicines onto formularies were recommendations from professional societies, evidence from the literature, results from the national tender and the price of the medicine (Plet et al., 2013). Similarly, Martin et al. (Martin et al., 2003) and Jenkings et al. (Jenkings and Barber, 2004) found in the UK that formulary decisions were based on a cluster of factors including the clinical benefits and the degree of certainty, with local knowledge also used in the UK (Martin et al., 2003; Jenkings and Barber, 2004). This is very different to the situation in some other sub-Saharan African countries where there is very variable PTC activities even among tertiary hospitals; however, this is not universal (Directorate of Pharmacy Services, 2012; Office of Director of Pharmaceutical Services (ODPS) Ministry of Health Ghana, 2015; Ashigbie et al., 2016; Mudenda et al., 2016; Fadare et al., 2018b; Anand Paramadhas et al., 2019).

Similarly, there were concerns with the extent of PTC activities among hospitals in rural Thailand, exacerbated by concerns with funding and lack of standard criteria for drug selection (Umnuaypornlert and Kitikannakorn, 2014). However, the review process was not uniform across all PTC hospital levels in our study despite the fact that the local EML has specific guidelines for the application for new medicines to be added to the EML and therefore the local formulary list. As seen, the standard information forms were typically not used among the different levels of hospital care in our study even though this is a requirement (Gauteng Province Department of Health, 2013; Meyer et al., 2017). This is a concern and in contrast to a study undertaken in Jordan where the majority (77%) of DTCs followed structured guidelines for formulary applications (Alefan et al., 2019).

The practice of lower level PTCs (district hospital and district PTCs) of only referring to the EML when reviewing level specific formularies was in accordance with Gauteng Provincial guidelines (Gauteng Province Department of Health, 2013), and in line with the findings of Perumal-Pillay and Suleman (Perumal-Pillay and Suleman, 2017). This was also influenced by the fact that at these levels, there is a limitation to the types of medicines that can be used due to a lack of specialized care. However, there is concern that PTC members among these hospitals lack expertise in EBM. This is important as physician’s trust in recommended medicines enhances their adherence to recommended medicines (Gustafsson et al., 2011; Bjorkhem-Bergman et al., 2013; Eriksen et al., 2017). The use of EMLs in formulary list reviews also took place in Thailand; however, there were concerns with defining the role of PTC members and monitoring outcomes of policies (Sudchada et al., 2012).

Cost considerations were important among all hospital levels both in terms of evaluating new more expensive medicines as well as alternative cheaper but equally effective medicines. This is in accordance with the Gauteng Provincial guidelines where if multiple medicines are equally clinically effective, cost considerations should be a key criteria (Gauteng Province Department of Health, 2013). This is in line with other countries (Mannebach et al., 1999; Eddama and Coast, 2008; Duran-Garcia et al., 2011; Gustafsson et al., 2011; Bjorkhem-Bergman et al., 2013), with typically indirect costs only considered in a minority of situations (Mannebach et al., 1999; Eddama and Coast, 2008). Cost considerations also resulted in restrictions on which patients should receive the new medicines especially in tertiary hospitals, similar to a number of European countries (Godman et al., 2012; Malmstrom et al., 2013; Parkinson et al., 2015). A key consideration certainly for tertiary hospitals was whether the local vetting committee had verified the costings in the application and made the necessary funds available (Gauteng Province Department of Health, 2013). The lack of pharmacoeconomic expertise certainly among tertiary hospitals should be borne in mind when considering formulary management in developing countries in the future (Matlala et al., 2017), as lack of expertise may lead to inappropriate use of medicines that are not cost effective (Pharasi and Miot, 2013). Co-opting health economics expertise if needed could be one way forward. However, this will depend on issues of affordability in LMICs who struggle to fund, for instance biological medicines for immune conditions as well as insulins and newer oral anti-diabetic medicines for patients with diabetes (Williams and Bryan, 2007; Putrik et al., 2014; Baumgart et al., 2019; Godman et al., 2020a; Godman et al., 2020b). The current lack of pharmacoeconomic expertise at the provincial level could potentially be addressed by the adoption of Health Technology Assessment (HTA) principles included in the NHI Bill (2018) (Meyer et al., 2017).

It is noteworthy that the use of EML/STGs among public sector hospitals in South Africa limits the degree to which clinicians can request medicines which do not appear on these documents. Having said this, it was encouraging especially in tertiary hospitals that acceptance of new medicines onto the formulary typically required the development of guidelines outlining their appropriate use. Laing et al. (2001) found that substantial involvement and consultation among end users enhanced the acceptability and usage of the guideline once available (Laing et al., 2001). We believe this demonstrates that PTC activity in South Africa can go further than dichotomous rulings by defining pertinent patient populations for the new medicine, similar to current practices in for instance Scotland, Slovenia, Spain and Sweden (Bjorkhem-Bergman et al., 2013; Godman et al., 2014a; Godman et al., 2015; Matusewicz et al., 2015; Eriksson et al., 2017). This makes the South Africa public sector restrictive, when compared to some developed countries. However, having clinical involvement and a limited list of medicines enhances adherence and quality of care through greater physician knowledge of agreed medicines as seen for instance in Stockholm, Sweden, with the ‘Wise List’ of medicines (Gustafsson et al., 2011; Bjorkhem-Bergman et al., 2013).

A concern is that even though some PTCs reviewed existing formulary lists based on medicine usage and availability, the process was not structured as most presentations were made verbally. However, this was similar to the study of Abdelrahim et al. (Abdelrahim et al., 2012), who reported that additions or removals of medicines were undertaken verbally in five (62.5%) hospitals (Abdelrahim et al., 2012). The lack of written documentation for requests to remove medicines from local formulary lists, i.e. disinvestment, can lead to confusion in future reviews as there would not be evidence to support such removals; consequently, lack of accountability. This has however been addressed by the National Department of Health in the form of a policy guideline instrument, published in 2019, which includes documentation to be completed when such requests are submitted to the PTC (South African National Department of Health, 2019).

Medicine utilization as a basis for removing, reviewing, or adding medicines to the formulary list, as observed in this study is encouraging similar to other countries and situations (Persson et al., 2013; Wehmeyer et al., 2019). However, there is a need to increase the number of drug utilization studies within hospitals to improve future medicine use and EBM skills. PTC activities could also be improved by hospitals sharing their experiences and expertise. We have seen such approaches used by health authorities across Europe when implementing policies to improve the use of resources without compromising care (Godman et al., 2014a; Godman et al., 2014b; Moon et al., 2014; Godman et al., 2019).

At the time this study was conducted, the only official national guiding document addressing the functioning of PTCs was the National Drug Policy (NDOH, 2015). Various provinces also had their own individual guidelines on the functions and structures of PTCs, such as the Gauteng Province (Gauteng. Gauteng Province Department of Health, 2013). In 2015 the first National Policy for the Establishment and Functioning of PTCs in South Africa was published (NDOH, 2015), which was then followed by the publication of the National Guideline for the Establishment and Functioning of Pharmaceutical and Therapeutics Committees in South Africa in 2019, providing the tools and practice guidance for the implementation of the policy (South African National Department of Health, 2019). This national guideline (South African National Department of Health, 2019) articulates the hierarchy for the formulary development and provides further guidance on other matters relating to formulary management nationally. These developments are important, as they will ensure uniformity in formulary management across the country.

We are aware of a number of limitations with our study. This included the fact that the study was undertaken in only one province out of possible nine in South Africa, which may impact the generalisability of the observations seen. This lack of generalisability of these results to the rest of the provinces in the country is influenced further by the autonomy of the various Provincial Departments of Health. The medicine selection decisions may vary from province to province, which may result in inequitable access to medicines and health care products (Pharasi and Miot, 2013). Gauteng Province is also the most populated and the economic hub of South Africa, therefore it has the highest number of central/specialized public sector hospitals in the country. In addition, PTC members may have been intimidated by the presence of an observer. We tried though to make observations as discreetly as possible so as to not influence the proceedings. However, in view of our methodology, we do believe our findings are robust providing direction for the future.

## Conclusions

We believe this is the first study conducted in the public healthcare sector in South Africa that has comprehensively looked at the processes followed by PTCs in their activities. PTCs at all levels of care were involved in formulary management, even though they all adopted different approaches depending on the level of care provided and the expertise of the committee. Principally, the review of local formularies for potential inclusion of new medicines was, as expected, guided by the current EML/STGs and the tertiary list. The approach for the potential inclusion of new medicines differed among the levels of care, with tertiary hospital PTCs, which cater for more specialized services such as oncology and transplant units, able to request medicines outside of the current EML/STGs. The decisions were typically based on EBM at the tertiary level and typically acquisition cost considerations at secondary and primary care hospitals. This reflects the greater freedom among tertiary hospitals to list new medicines not included in the standard EML. The PTCs in Gauteng might not have had more freedom to include medicines outside of the EML or the tertiary list, but they still applied the principles of formulary development, observed in the private sector and other developed countries.

However, a number of concerns were identified. These included limited utilization of standard forms for requests for new medicines to be included in the formularies, limited use of EBM principles for formulary considerations and currently limited use of drug utilization studies to monitor the subsequent use of medicines following decisions as well as any impact on patient care in routine clinical practice. There was also an identified need for PTCs to learn from each other. In addition, there was a lack of health economic considerations in formulary listing decisions. These considerations could become more holistic with recent HTA proposals; though, this remains to be seen.

## Data Availability Statement

The raw data supporting the conclusions of this article will be made available by the corresponding author, without undue reservation.

## Ethics Statement

The study received ethical clearance from the Medunsa Research Ethics Committee, University of Limpopo (Medunsa Campus) and approval from the Gauteng Provincial Department of Health Research Committee. The respective PTC chairpersons provided permission to record proceedings, and where permission for recording was not provided, notes of proceedings were taken.

## Author Contributions

MM, AG, and JM contributed to the conceptualization and design of the study, and the interpretation of the findings. MM collected the data. MM and BG undertook the literature review. All authors contributed to the article and approved the submitted version.

## Funding

MM received a research and development grant from the University of Limpopo (Medunsa Campus).

## Conflict of Interest

The authors declare that the research was conducted in the absence of any commercial or financial relationships that could be construed as a potential conflict of interest.
